# Involvement of the DNA Phosphorothioation System in TorR Binding and Anaerobic TMAO Respiration in Salmonella enterica

**DOI:** 10.1128/mbio.00699-22

**Published:** 2022-04-14

**Authors:** You Tang, Yue Hong, Li Liu, Xuan Du, Yaqi Ren, Susu Jiang, Wanjing Liu, Chen Chao, Zixin Deng, Lianrong Wang, Shi Chen

**Affiliations:** a Ministry of Education Key Laboratory of Combinatorial Biosynthesis and Drug Discovery, Hubei Clinical Center and Key Laboratory of Intestinal and Colorectal Disease, Department of Gastroenterology, Zhongnan Hospital of Wuhan University, School of Pharmaceutical Sciences, Wuhan University, Wuhan, Hubei, China; b Department of Burn and Plastic Surgery, Shenzhen Institute of Translational Medicine, Health Science Center, The First Affiliated Hospital of Shenzhen University, Shenzhen Second People’s Hospital, Shenzhen, China; New York University School of Medicine

**Keywords:** DNA phosphorothioate modification, *tor* boxes, competitive binding

## Abstract

Although the phosphorothioate (PT) modification, in which the nonbridging oxygen in the DNA sugar-phosphate backbone is replaced by sulfur, has been reported to play versatile roles in multiple cellular processes, very little data have been obtained to define the role of PT in epigenetic regulation. In this study, we report that the PT system in Salmonella enterica serovar Cerro 87 is involved in the transcriptional regulation of the *torCAD* operon encoding the trimethylamine N-oxide (TMAO) respiration machinery that enables the use of TMAO as a terminal electron acceptor for respiration when oxygen is not available. *In vitro*, PT enhanced the binding of the transcriptional activator of the *torCAD* operon, namely, TorR, to its DNA substrate (*tor* boxes). However, *in vivo*, the PT modification protein complex DndCDE downregulated *torCAD* transcription through competing with the binding of TorR to the *tor* boxes. The altered expression of *torCAD* caused by PT modification proteins affected cell growth that relied on TMAO respiration. To our knowledge, this is the first report supporting that PT proteins participate in transcriptional regulation, showing a new function of PT systems.

## INTRODUCTION

Epigenetic control of gene expression is considered an exclusive function of DNA modifications (1). The presence of 5-methylcytosine (5mC) in the context of CpG dinucleotides can regulate eukaryotic gene transcription by impairing transcription factor binding or recruiting repressive methyl-binding proteins (2, 3). For instance, a large proportion of selfish genetic elements, i.e., transposons, retrotransposons, and viral elements, in the mammalian genome are silenced by 5mC (4, 5). Another example of a critical transcription-regulating DNA modification in eukaryotes is 5-hydroxymethylcytosine (5hmC), which was recently confirmed to be prevalent in embryonic stem cells and in the mammalian brain and has been reported to regulate gene expression during embryonic development and the function of the brain (6–8).

In prokaryotes, DNA methylation governed by methyltransferases (MTases) is typically coupled with a restriction endonuclease (REase) and functions as a restriction (R)-modification (M) system to provide protection against incoming foreign DNA (9). In contrast, a solitary MTase has no REase companion to defend against invaders but is nevertheless involved in regulating gene expression (10, 11). Examples of solitary MTases include DNA adenine methyltransferase (Dam) and cell cycle-regulated methylase (CcrM), which methylate adenine at the N6 position at the 5′-GATC-3′ and 5′-GANTC-3′ sites, respectively (10–12). A variety of genes have been reported to be under the epigenetic control of N^6^-methyladenine, e.g., *tnp* (*IS10*), *traJ*, *dnaA*, *ctrA*, *papBA*, *sciH*, and *opvAB* (13–19). These genes are involved in diverse physiological processes, including transposition, plasmid transfer, DNA replication, cell cycle control, fimbria production, type VI secretion, and lipopolysaccharide modification, substantially expanding the breadth of DNA methylation functions to include more than R-M defense. Generally, bacterial DNA methylation modulates gene transcription by interfering with protein–DNA interactions. It can promote or inhibit the binding of RNA polymerase or transcription factors to their DNA substrates (20, 21).

Recent explorations into DNA structure have facilitated the discovery of previously unknown DNA modifications, such as the phosphorothioate (PT) modification (22). In the PT modification of DNA, the nonbridging oxygen in the DNA sugar-phosphate backbone is replaced by sulfur (22, 23). Typically, a double-stranded PT modification is mediated by *dndABCDE* gene products and occurs in a sequence-selective and *R*_P_ stereospecific manner (22, 24–27). DndA and DndCDE are essential to confer the DNA PT modification, and DndB functions as a transcriptional repressor of the *dndBCDE* operon (25, 28). Although the precise process of sulfur incorporation is not fully understood, some clues have been elucidated to date: DndA is a cysteine desulfurase that catalyzes the removal of the S atom from l-cysteine, generating l-alanine and a protein-bound cysteine persulfide intermediate (R-S-SH) (29, 30). Persulfide is a common sulfur donor for the subsequent biosynthesis of Fe-S clusters, thiamine, thionucleosides in tRNA, biotin, etc. (31). Interestingly, DndC contains a 4Fe-4S cluster, and DndA catalyzes the assembly of the Fe-S cluster in DndC (29). DndD shows high homology to structural maintenance of chromosome (SMC) proteins, and DndE preferentially binds to nicked DNA (32, 33). IscS (a DndA homolog), DndC, DndD, and DndE have been observed to form a complex at an approximate molecular ratio of 1:1:1:1 *in vitro* (34). A recent study showed that DndCDE-IscS confers the DNA PT modification in the presence of SAM, Mg^2+^, ATP, and cysteine, supporting the PT reaction through a radical SAM mechanism (35). All these observations suggest that sulfur is first transferred from cysteine to DndA and then to the cysteine residues in DndC before it is ultimately incorporated into the DNA backbone through the coordinated functions of DndD and DndE (36).

Phylogenetic analysis has revealed that *dnd* genes are distributed in more than 2,000 bacterial and archaeal strains (37, 38). In some bacteria, DndABCDE are coupled with DndFGH, SspE, PbeABCD, or SspFGH to confer resistance to invading foreign DNA (38–41), functionally resembling the DNA methylation-based R-M defense barrier. In contrast, some Dnd systems occur as bona fide solitary DndABCDE systems lacking restriction cognates (37). In addition to being a component in PT-based defense systems, studies have shown that the DNA PT modification has evolved to play versatile roles in antioxidant defenses, cellular redox homeostasis maintenance, environmental stress resistance, and cross talk with the DNA methylation modification (26, 37–40, 42–44).

According to one recent report, PT modification of the *fpsR*-*fpsA* intergenic region increases the binding of the phage-borne repressor FpsR and decreases the amount of FpsR protein, thereby relieving the repression of phage gene transcription (45). In addition, several observations support the capacity of PT to interfere with protein–DNA interactions: (i) the presence of PT in DNA templates is capable of altering transcription by RNA polymerase *in vitro* (37) and (ii) PT modification at 5′-GATC-3′ (generating 5′-G_PS_ATC-3′) slows Dam-catalyzed methylation at this site (44). However, very little data on the ability of PT to modulate bacterial gene transcription have been reported thus far.

In this study, we investigated whether the PT modification interfered with certain transcription factor-DNA interactions and, thus, regulated gene expression. By performing a pulldown assay coupled with proteomic quantification, we found that the PT modification of the 5′-G_PS_AAC-3′/5′-G_PS_TTC-3′ motif enhanced the binding of TorR, the transcriptional activator of the *torCAD* operon, to a 30-bp DNA probe. Surprisingly, the transcription of the *torCAD* operon was downregulated *in vivo* due to the competitive binding of Dnd proteins to the natural TorR binding sites, termed *tor* boxes. Consequently, bacterial growth was attenuated during anaerobic respiration using trimethylamine N-oxide (TMAO) as the terminal electron acceptor. These findings showed that the PT modification alters the binding of certain bacterial transcription factors to DNA substrates and that PT modification proteins are also involved in transcriptional regulation and, thus, play a role in bacterial anaerobic TMAO respiration.

## RESULTS

### PT modification of the 5′-G_PS_AAC-3′/5′-G_PS_TTC-3′ motif enhanced the binding of TorR to *tor* boxes.

We have previously shown that the presence of PT in DNA templates alters transcription by RNA polymerase *in vitro* (37), suggesting an epigenetic regulatory function of the PT modification under physiological conditions. In this study, we performed pulldown assays using biotinylated DNA probes and the cell lysate of Salmonella enterica serovar Cerro 87 that contains the *dndBCDE-dndFGH* module to capture proteins that exhibit differential binding to PT- and non-PT-modified DNA *in vivo*. For these experiments, we used two 30-bp DNA probes, B12 and B34, which share the same DNA sequences but possess PT-modified 5′-G_PS_AAC-3′/5′-G_PS_TTC-3′ and non-PT-modified 5′-GAAC-3′/5′-GTTC-3′ motifs, respectively (see Table S1 in the supplemental material). By coupling the pulldown assay with mass spectrometry, we identified 12 proteins that showed greater than 2-fold differences in binding affinities (*P* < 0.05) for the B12 and B34 probes (Table 1). Seven proteins, DndC, DndF, DndG, the d-serine dehydratase transcription activator DsdC, the *torCAD* operon transcriptional regulatory protein TorR, phosphopentomutase, and the helicase PriA, exhibited stronger binding affinity for the PT-modified B12 probe. In contrast, five proteins, dGTP triphosphohydrolase, the exodeoxyribonuclease VII large subunit, the ATP-dependent RNA helicase RhlB, the biotin carboxyl carrier protein of acetyl-coenzyme A (CoA) carboxylase, and the formate hydrogenlyase transcription activator FhlA, showed higher binding affinity for the non-PT-modified B34 probe (Table 1 and Data Set S1).

**TABLE 1:** Proteins pulled down using PT-modified or non-PT-modified oligonucleotides with fold changes greater than 2^*a*^

ID of proteins detected using LC-MS/MS	No. of unique peptide MS/MS spectrum			No. of unique peptide MS/MS spectrum			Non-PT-modified (B34)/PT-modified (B12) ratio	*P* value	Function
	B12-I	B12-II	Avg after normalization	B34-I	B34-II	Avg after normalization			
GW13_PRO2829	6	8	6.57	0	0	0.00	0.00	0.020*	TorCAD operon transcriptional regulatory protein (TorR)
GW13_PRO2800	17	16	15.47	3	1	1.94	0.13	0.006**	d-Serine dehydratase transcriptional activator
GW13_PRO3082	6	8	6.57	2	1	1.46	0.22	0.039*	Helicase PriA essential for *oriC*/DnaA-independent DNA replication
GW13_PRO3441	34	32	30.94	11	9	9.76	0.32	0.004**	DNA phosphorothioation-dependent restriction protein DndG
GW13_PRO3517	4	4	3.75	1	2	1.48	0.39	0.038*	Phosphopentomutase
GW13_PRO3446	6	7	6.10	3	2	2.44	0.40	0.030*	3′-Phosphoadenosine 5′-phosphosulfate sulfurtransferase DndC
GW13_PRO3440	21	22	20.16	11	7	8.76	0.43	0.026*	DNA phosphorothioation-dependent restriction protein DndF
GW13_PRO1601	10	7	7.96	18	17	17.11	2.15	0.030*	Exodeoxyribonuclease VII large subunit
GW13_PRO2912	6	9	7.04	16	16	15.66	2.22	0.030*	ATP-dependent RNA helicase RhlB
GW13_PRO2390	1	2	1.41	4	4	3.91	2.78	0.038*	Biotin carboxyl carrier protein of acetyl-CoA carboxylase
GW13_PRO3771	4	3	3.28	12	12	11.74	3.58	0.003**	Deoxyguanosine triphosphate triphosphohydrolase; subgroup 1
GW13_PRO1832	0	0	0	2	3	2.46	5.24	0.038*	Formate hydrogenlyase transcriptional activator

a I and II represent two independent samples analyzed using the pulldown assay. *, *P* < 0.05; **, *P* < 0.01; 0, not detected.

10.1128/mbio.00699-22.2 TABLE S1 Primer sequences used in this study. Download Table S1, DOCX file, 0.03 MB. Copyright © 2022 Tang et al. 2022 Tang et al. https://creativecommons.org/licenses/by/4.0/ This content is distributed under the terms of the Creative Commons Attribution 4.0 International license.

10.1128/mbio.00699-22.4 DATA SET S1 Proteins pulled down with PT-modified or non-PT-modified oligonucleotides. Download Data Set S1, XLSX file, 0.1 MB. Copyright © 2022 Tang et al. 2022 Tang et al. https://creativecommons.org/licenses/by/4.0/ This content is distributed under the terms of the Creative Commons Attribution 4.0 International license.

Three regulatory proteins, FhlA, DsdC, and TorR, attracted our attention because their specific interactions with PT-containing DNA substrates would likely enable PT to modulate transcription under physiological conditions. FhlA and DsdC are transcriptional activators of the formate hydrogenlyase system and d-serine detoxification locus, respectively (46–49). However, a 5′-GAAC-3′/5′-GTTC-3′ consensus was not identified in either the FhlA- or DsdC-binding sites, suggesting that the PT modification is unlikely to be involved in their regulatory functions *in vivo* by affecting their interactions with regulatory regions. As FhlA activates transcription of genes, including *fdhF*, and DsdC activates transcription of the *dsdXA* operon, we fused the promoter sequences of *fdhF* and *dsdXA* to the promoterless *lacZ* gene to assess FhlA- and DsdC-mediated transcription in wild-type Cerro 87 and *dndBCDE-dndFGH*-lacking mutant XTG103 (Table S2), respectively, as a method to test this hypothesis. The immediate observation was that the P*_fdhF_*-*lacZ* and P*_dsdXA_*-*lacZ* fusions displayed negligible changes in expression regardless of the presence of the Dnd system, as determined by measuring β-galactosidase activity (Fig. S1). This result suggested that the regulatory activities of FhlA and DsdC are not sensitive to the DNA PT modification, DndBCDE or DndFGH proteins.

10.1128/mbio.00699-22.1 FIG S1 Beta-galactosidase activity of P*_fdhF_* (A) and P*_dsdXA_* (B) in the Cerro 87 and XTG103 strains. Download FIG S1, TIF file, 1.1 MB. Copyright © 2022 Tang et al. 2022 Tang et al. https://creativecommons.org/licenses/by/4.0/ This content is distributed under the terms of the Creative Commons Attribution 4.0 International license.

10.1128/mbio.00699-22.3 TABLE S2 Strains and plasmids used in this study. Download Table S2, DOCX file, 0.02 MB. Copyright © 2022 Tang et al. 2022 Tang et al. https://creativecommons.org/licenses/by/4.0/ This content is distributed under the terms of the Creative Commons Attribution 4.0 International license.

**FIG 1:**
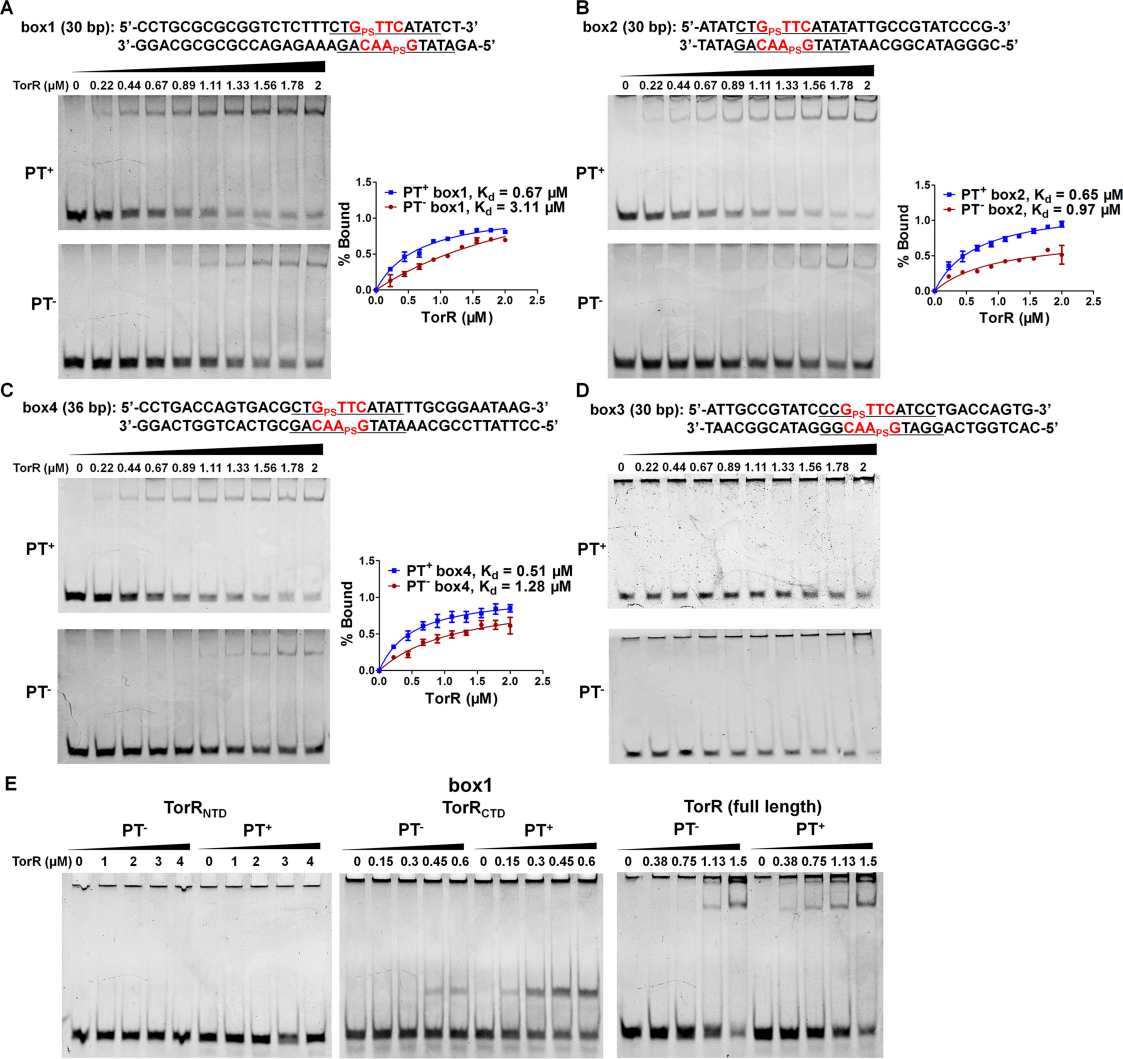
Effect of the DNA PT modification on TorR binding. DNA fragments of 50 nM covering 5′-G_PS_TTC-3′/5′-G_PS_AAC-3′-modified (upper) or nonmodified (lower) *tor* box1 (A), box2 (B), box4 (C), or box3 (D) were incubated with increasing concentrations of TorR in 20 μL of binding buffer (20 mM Tris-HCl, pH 8.0, 50 mM KCl, 10 mM MgCl_2_, 5 mM EDTA, 10 mM DTT, and 5% glycerol) and then subjected to 9% polyacrylamide gel electrophoresis. DNA sequences are displayed with 5′-G_PS_TTC-3′/5′-G_PS_AAC-3′ motifs colored in red and *tor* boxes 1, 2, 3, and 4 underlined. The intensity of the shifted DNA band is reported as the percent bound. Data are shown as the means ± standard deviations (SD) from three independent experiments. (E) PT-modified or non-PT-modified *tor* box1 was incubated with increasing concentrations of TorR_NTD_, TorR_CTD_, or full-length TorR and then resolved on EMSA gels. Gel electrophoresis was performed at 4°C and 160 V for 45 min.

Notably, the DNA binding region, consisting of box1, box2, and box4 (5′-CTGTTCATAT-3′) and box3 (5′-CCGTTCATCC-3′), of TorR, the transcriptional activator of the *torCAD* operon encoding the TMAO reductase respiratory system in response to anaerobic conditions in the presence of TMAO, harbors four 5′-GTTC-3′/5′-GAAC-3′ motifs, indicating PT-mediated transcriptional interference *in vivo* (50, 51). Because the probe sequence used in the pulldown assay matched only six and five of the 10 consensus nucleotides in boxes 1, 2, and 4 and in box3, respectively, a quantitative electrophoresis mobility shift assay (EMSA) was performed using TorR with 5′-G_PS_TTC-3′/5′-G_PS_AAC-3′ containing natural TorR binding sites (boxes 1 to 4). As shown in Fig. 1, TorR bound PT-modified boxes 1, 2, and 4 with dissociation equilibrium constant (*K_d_*) values of 0.67, 0.65, and 0.51 μM, respectively, which were lower than those of 3.11, 0.97, and 1.28 μM measured with corresponding non-PT-modified *tor* boxes (Fig. 1A to C). This result suggested that TorR bound more tightly to PT-modified *tor* boxes 1, 2, and 4, consistent with the results of the pulldown assay. Despite the presence of the PT modification, no retarded DNA band was observed after the incubation of the box3 region with TorR protein (Fig. 1D). This finding is reasonable because box3 harbors a low-affinity binding site matching only 7 of the 10 typical *tor* box nucleotides (boxes 1, 2 and 4: 5′-CTGTTCATAT-3′ (typical tor box sequences), box3: 5′-CCGTTCATCC-3′, underlined sequences show the 7 nucleotides that match the typical *tor* box sequences), and *in vitro* TorR-box3 binding likely depends on prior binding at the box4 site (52). TorR belongs to a subfamily of response regulators of two-component systems (TCSs). Proteins in this subfamily generally consist of an N-terminal regulatory domain and a C-terminal DNA-binding domain (53). To exclude the possibility that the PT-binding preference resulted from nonspecific interactions, we purified the individual N-terminal (M1-R131) and C-terminal (P139-Y242) domains of TorR and tested their binding with box1. A retarded band was observed when the C-terminal domain (CTD) was assessed (Fig. 1E). In contrast, no such band emerged when the N-terminal domain (NTD) was assayed, regardless of the presence of the PT modification (Fig. 1E). Again, similar to the full-length TorR, TorR_CTD_ exhibited stronger binding affinity for PT-DNA, confirming that the preferential PT binding was attributed to the CTD of TorR (Fig. 1E).

### Transcription of the *torCAD* operon *in vivo* is repressed in *dnd*-containing cells and is sensitive to DndCDE proteins but not to the DNA PT modification.

**FIG 2:**
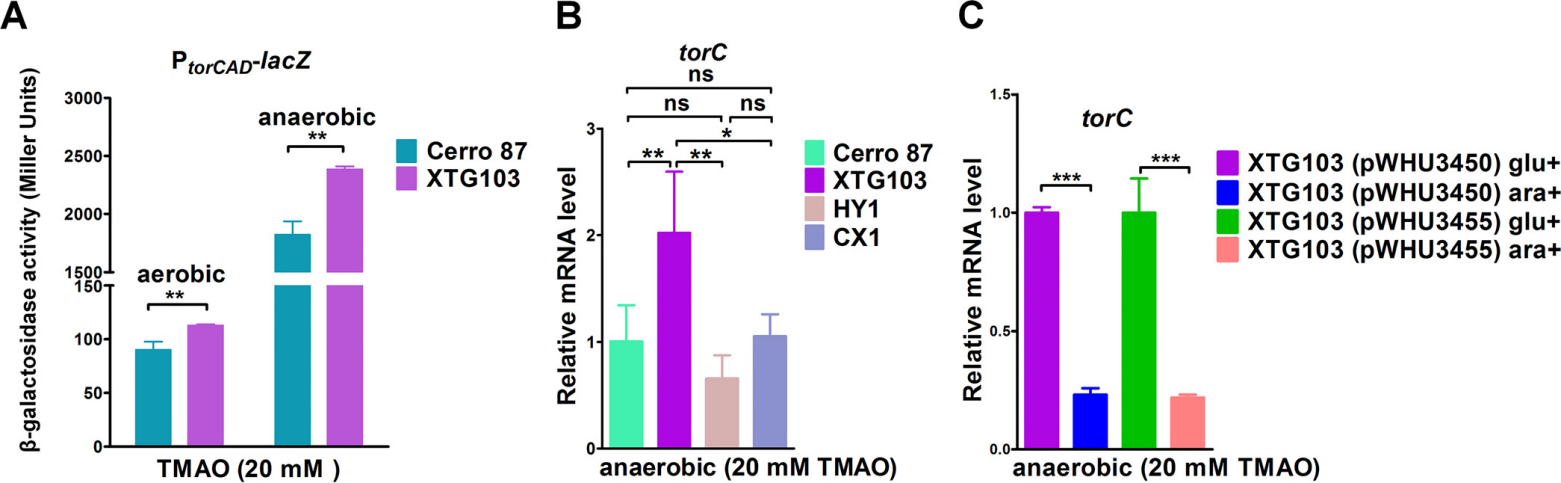
Analysis of TorR-activated transcription of *torCAD* in wild-type Cerro 87 and PT-deficient cells. (A) The promoter region of the *torCAD* operon was fused with the promoterless *lacZ* reporter gene and cloned into the pACYC184 plasmid. β-Galactosidase activity, which is reported in Miller units, was measured to determine the promoter activity in Cerro 87 and XTG103 cells under aerobic and anaerobic conditions, respectively. (B) The relative *torC* mRNA level in strains Cerro 87, XTG103, HY1, and CX1. (C) qRT-PCR analysis of the relative *torC* mRNA levels in different strains; *gapA* was used as a reference gene. In addition, 0.4% glucose (glu) or 0.4% arabinose (ara) was added to repress or induce expression of the DndCDE or DndC_C280S_DE protein, respectively, from cloned plasmids in anaerobically cultivated strains. The data represent three independent experiments. *, *P* < 0.05; **, *P* < 0.01; ***, *P* < 0.001; ns, not significant.

The PT-binding preference prompted us to hypothesize that TorR-dependent *torCAD* expression would be increased in PT-containing cells in the presence of TMAO. However, the β-galactosidase activity of the P*_torCAD_*-*lacZ* fusion construct was 1.31-fold higher in *dndBCD-dndFGH*-lacking XTG103 cells than in wild-type Cerro 87 cells grown under anaerobic conditions (Fig. 2A), suggesting that TorR binding and promoter activation of the *torCAD* operon were repressed rather than enhanced by the PT-based Dnd system *in vivo*. This finding was reinforced by the observation that the level of the *torC* transcript increased by 2.0-fold upon the deletion of the *dndBCDE-dndFGH* module in XTG103 (Fig. 2B). The contradiction between the *in vivo* and *in vitro* results prompted us to construct a mutant strain, HY1, expressing the chromosomal *dndBCDE* operon in which a single cysteine residue (C280) was replaced with a serine residue in DndC. The single-point C280S mutation completely abolished the DNA PT modification (35), but the complete DndCDE protein complex was retained. Interestingly, despite the loss of the DNA PT modification, *torC* in the HY1 strain displayed the same transcription level as that in wild-type Cerro 87 (Fig. 2B). Together with the observation that (i) the deletion of the *dndFGH* operon did not cause a disinhibitory effect on the transcription of *torC* in the CX1 mutant and (ii) overexpression of DndCDE or DndC_C280S_DE in XTG103 remarkably inhibited the transcription of *torC*, we concluded that TorR-dependent *torCAD* operon expression *in vivo* was susceptible to DndCDE proteins rather than the DNA PT modification (Fig. 2B and C). We suspect that the 5′-GTTC-3′/5′-GAAC-3′ motifs embedded in the *tor* boxes would probably be the recognition targets of Dnd proteins; therefore, they would competitively bind to the promoter region of the *torCAD* operon, resulting in a lower transcriptional level.

### DndCDE and TorR bind competitively to the *tor* regulatory region.

**FIG 3:**
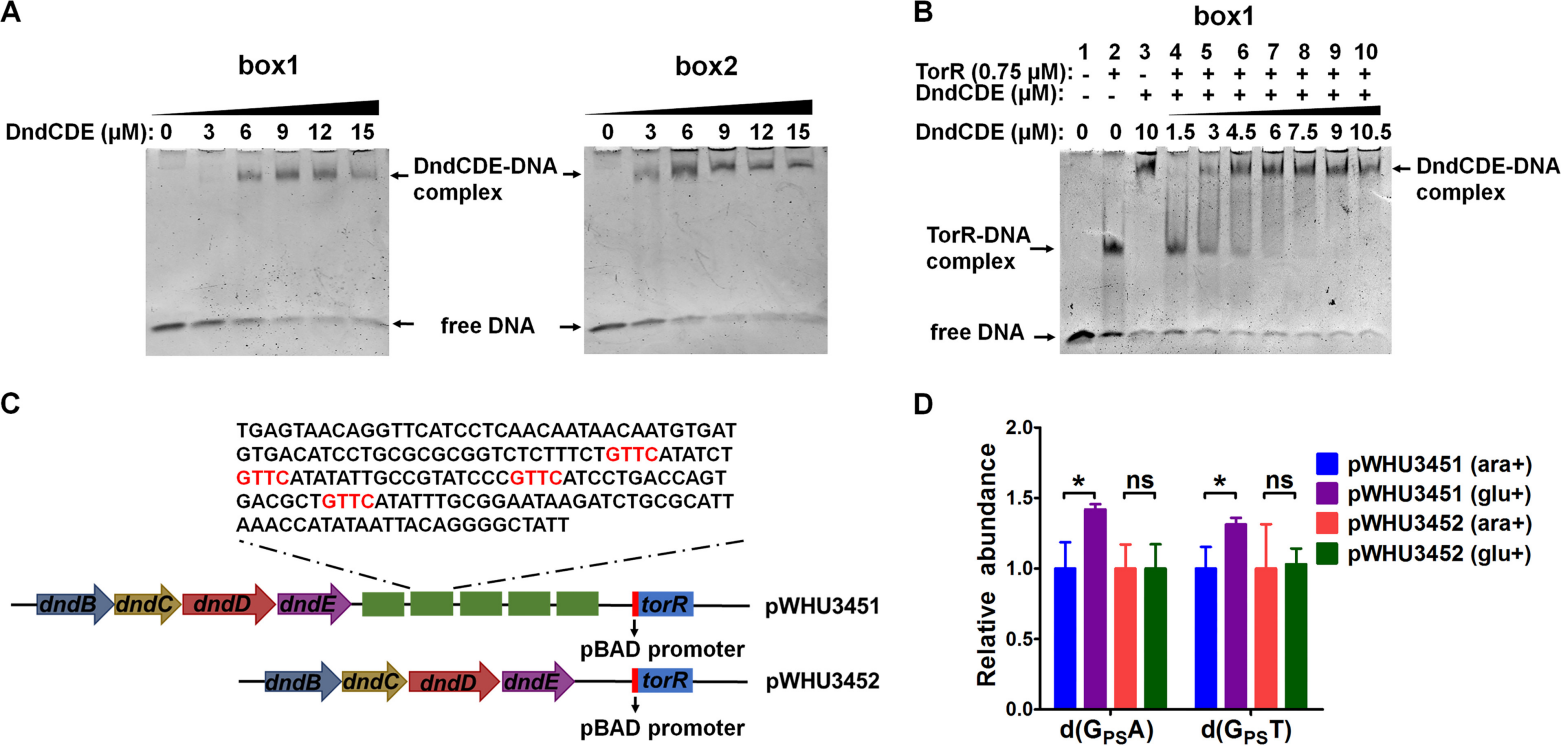
Competitive binding of DndCDE and TorR to the regulatory region of the *torCAD* operon. (A) For this experiment, 50 nM DNA fragments containing *tor* box1 or box2 were incubated with increasing concentrations of DndCDE complex and then subjected to 4% polyacrylamide gel electrophoresis. (B) When 50 nM *tor* box1-containing DNA fragment was incubated with 0.75 μM TorR, retardation due to DNA-TorR complex formation was observed (lane 2), which was competed with increasing concentrations of DndCDE protein complex (lanes 4 to 10). (C) The expression of the *torR* gene in both the pWHU3451 and pWHU3452 plasmids was under the control of the pBAD promoter that is inducible with arabinose. pWHU3451 contains a fragment consisting of five repeats of the *tor* regulatory region, while pWHU3452 does not. (D) The induction of TorR expression reduced PT abundance in plasmid pWHU3451 DNA. E. coli DH10B harboring pWHU3451 or pWHU3452 was anaerobically grown in LB supplemented with 20 mM TMAO, and 0.4% glucose (glu) or 0.4% arabinose (ara) was added to repress or induce the expression of TorR. The abundances of d(G_PS_A) and d(G_PS_T), which are enzymatically released from plasmid DNA, were quantified using LC-MS/MS. The data represent three independent experiments. *, *P* < 0.05; ns, not significant.

To determine whether DndCDE competed with TorR for binding to the *tor* regulatory region, we first performed EMSAs using boxes 1 and 2 with DndCDE. As shown in Fig. 3A, DndCDE bound these boxes. We then assessed the DNA retardation pattern of TorR in the presence of DndCDE by performing a competitive gel mobility shift assay. As shown in Fig. 3B, when the TorR concentration remained constant, the addition of increasing concentrations of DndCDE profoundly inhibited TorR-DNA binding. If DndCDE and TorR could simultaneously bind the DNA substrate, then a DNA-TorR-DndCDE complex with a higher molecular weight would form. However, no such apparent complex was observed, indicating that DndCDE and TorR bind competitively to the *tor* regulatory region. This result explains the finding that TorR-dependent *torCAD* expression was inhibited in *dnd*-expressing Salmonella cells.

Considering that DndCDE and TorR competitively bind *tor* regulatory sequences, we hypothesized that this competition alters the access of DndCDE at this region to generate PT. To test this hypothesis, we constructed the plasmid pWHU3451, which harbored *torR* under the arabinose-inducible promoter pBAD and five repetitive *tor* regulatory regions (from box1 to box4) (Fig. 3C). The introduction of the *tor* box sequences resulted in 20 additional 5′-GAAC-3′/5′-GTTC-3′ motifs in pWHU3451, allowing us to assess the effect of TorR on DNA PT formation. When TorR expression was induced by 0.4% arabinose in Escherichia coli, the total PT level of d(G_PS_A) and d(G_PS_T) (PS, phosphate-sulfur linkage) in pWHU3451 decreased by 25% (Fig. 3D). In contrast, a similar PT alteration was not observed in pWHU3452 (Fig. 3D), which is a pWHU3451 derivative that lacks the five repetitive *tor* boxes (Fig. 3C). These results showed that the 5′-GTTC-3′/5′-GAAC-3′ motifs in *tor* boxes were occluded by overexpressed TorR and subsequently blocked DndCDE access to these recognition sites to generate PT-modified 5′-G_PS_TTC-3′/5′-G_PS_AAC-3′.

### Competitive interaction of DndCDE and TorR at the *tor* regulatory region alters TMAO anaerobic respiration.

**FIG 4:**
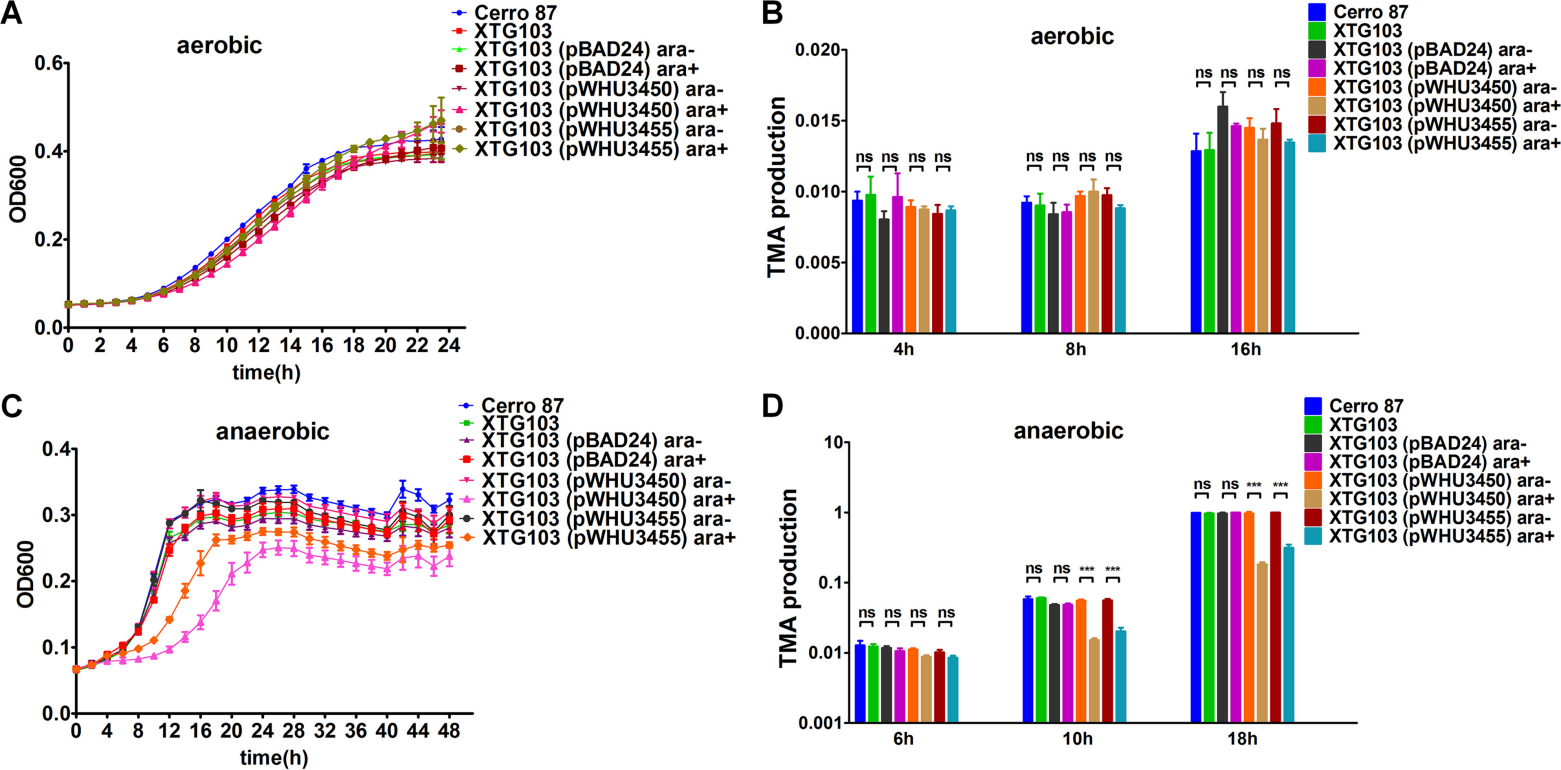
Growth of strains that rely on TMAO respiration and their TMAO metabolic rate detected using LC-MS/MS. Growth of Cerro 87, XTG103, XTG103 (pBAD24), XTG103 (pWHU3450), and XTG103 (pWHU3455) strains in modified M9 medium (0.4% glycerol was used as the carbon source and cultures were supplemented with 20 mM TMAO) under aerobic conditions (A) and anaerobic conditions (C). The production of TMA under aerobic (B) and anaerobic conditions (D) was measured using LC-MS/MS, as described in Materials and Methods. ara+, arabinose added; ara−, arabinose not added. The data represent three independent experiments. ***, *P* < 0.001; ns, not significant.

**FIG 5:**
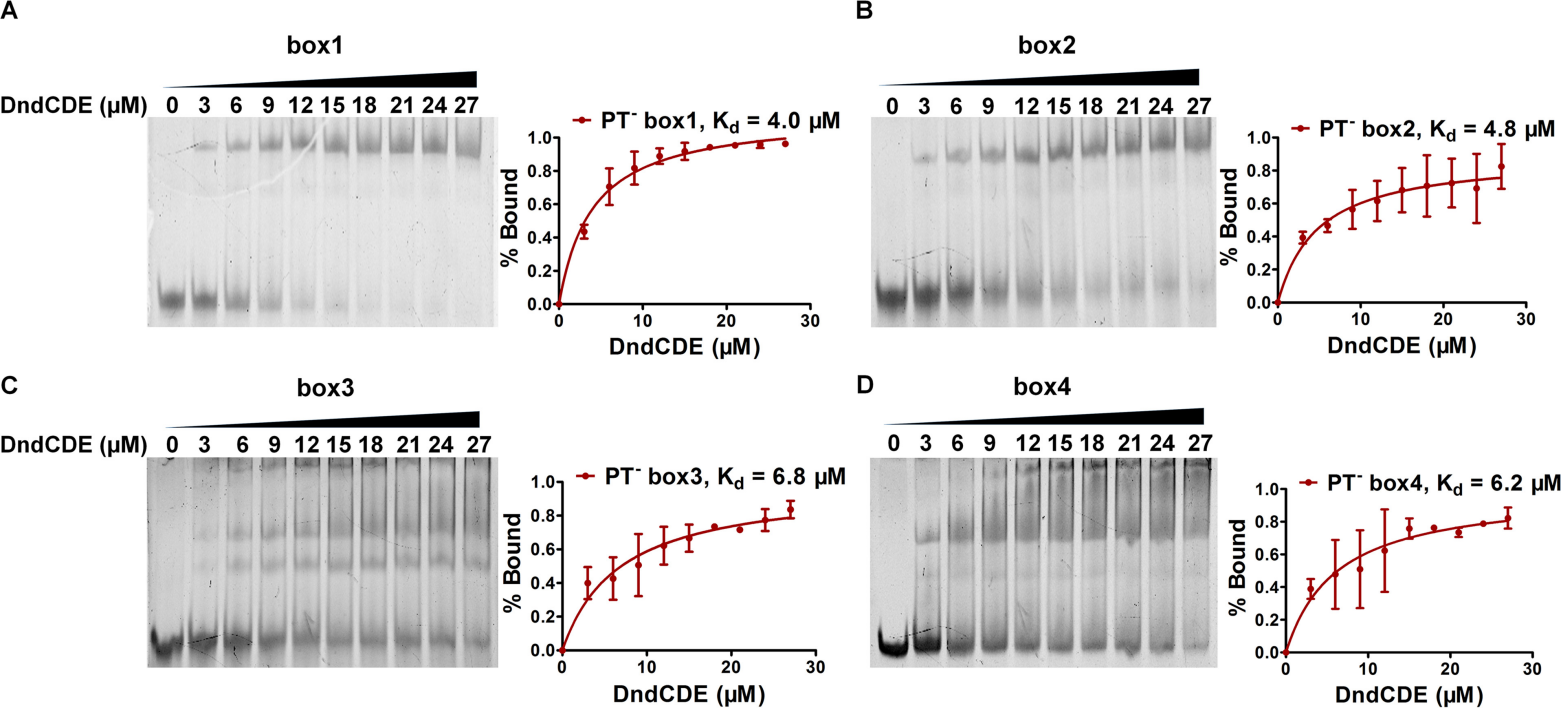
Binding of DndCDE to *tor* boxes. DNA fragments covering non-PT-modified *tor* box1 (A), box2 (B), box3 (C), or box4 (D) at a concentration of 50 nM were incubated with increasing concentrations of DndCDE at 28°C for 30 min, and the samples were then electrophoretically separated on 4% polyacrylamide gels. The intensity of the shifted DNA band is reported as the percent bound.

In the absence of oxygen, bacteria can use other small compounds, i.e., nitrate, nitrite, dimethyl sulfoxide, TMAO, and fumarate, as terminal electron acceptors for respiration (54). Anaerobic respiration of these compounds activates more energy-efficient metabolic pathways than fermentation alone and, thus, provides a significant fitness advantage for anaerobic adaptation (55). In the TMAO respiration pathway, the sensor protein TorS detects TMAO and transphosphorylates TorR, which in turn induces *torCAD* expression (56). The products of the *torCAD* gene use TMAO as the terminal electron acceptor to produce energy and support cell growth. The competitive interaction of DndCDE and TorR at the *tor* regulatory region prompted us to assess the growth of Cerro 87 and XTG103 dependent on TMAO respiration. Despite Dnd protein expression in Cerro 87 and the resulting transcriptional repression of the *torCAD* operon, Cerro 87 and XTG103 exhibited the same growth profiles under both aerobic and anaerobic conditions (Fig. 4A and C). These findings reminded us of the previous single-molecule real-time (SMRT) sequencing results for all PT-modified sites in the genome that revealed a relative underrepresentation of T and G residues preceding and A residues following the PT-modified 5′-G_PS_TTC-3′ motif, respectively (57). Notably, *tor* box1, box2, and box4 harbor 5′-tGTTCa-3′ motifs that are typically PT modified at a very low level (57). One plausible explanation is that TorR exhibits stronger binding affinity for *tor* regulatory regions and subsequently obviates Dnd proteins. To test this hypothesis, we determined the binding affinities of TorR and DndCDE for *tor* boxes by performing gel shift assays. TorR and DndCDE bound to *tor* boxes 1, 2, and 4 with *K_d_* values of 0.97 to 3.11 μM and 4.0 to 6.2 μM, respectively (Fig. 1 and 5), consistent with the SMRT sequencing and growth results. However, growth retardation of XTG103 (pWHU3450) and XTG103 (pWHU3455) in the presence of TMAO was detected when DndCDE and DndC_C280S_DE were overexpressed under anaerobic conditions, indicating a Dnd protein-mediated effect on gene transcription (Fig. 4C).

In addition, to monitor the TMAO respiration rate of strains more directly, we established a liquid chromatography with tandem mass spectrometry (LC-MS/MS) method to quantify the levels of TMAO and its reduced product, TMA. Similar to the growth results, the TMAO metabolic rate of XTG103 was not higher than that of Cerro 87 (Fig. 4B and D), although the expression level of the *torCAD* operon was higher than that in Cerro 87. We consider that the 1.31-fold higher expression of *torCAD* in XTG103 caused a change in TMAO metabolism that may have been difficult to detect using our LC-MS/MS analysis. Indeed, when the Dnd protein was overexpressed, we observed a much slower TMAO metabolic rate under anaerobic conditions (Fig. 4D), consistent with the growth result and supporting the hypothesis that Dnd proteins downregulated *torCAD* transcription to repress anaerobic TMAO respiration.

## DISCUSSION

Considering the key role of DNA modifications in epigenetic regulation, we questioned whether the DNA PT modification interferes with protein–DNA interactions, thereby regulating gene expression. By performing pulldown assays coupled with proteomic quantification, we identified three regulatory proteins, i.e., DsdC, TorR, and FhlA, that showed different binding affinities for PT-modified and non-PT-modified DNA. In addition, proteins with various functions, such as dGTP triphosphohydrolase and the ATP-dependent RNA helicase RhlB, were also sensitive to the PT modification, suggesting that PT modifications serve as signals for relevant physiological processes. More PT modification- and restriction-related proteins, e.g., DndC, DndF, and DndG, were pulled down by the PT-modified B12 DNA substrate than by the unmodified counterpart B34, suggesting that these Dnd proteins are involved in site recognition during sulfur incorporation and restriction processes.

In DNA methylation, if a methylation site embedded in a promoter or a regulatory region modulates the binding of RNA polymerase or transcription factors, it regulates gene expression (10, 20, 21). Based on this information, we were surprised to find that the binding sites of the transcriptional activator TorR contain a 5′-GAAC-3′/5′-GTTC-3′ motif (57), indicating the likelihood that PT regulates TorR-dependent *torCAD* operon transcription. Our results revealed that PT modulated *torCAD* transcription in unexpected ways: *in vitro*, PTs at 5′-GAAC-3′/5′-GTTC-3′ sites in the *torCAD* regulatory region enhanced TorR binding; *in vivo*, unexpectedly, *torCAD* transcription was repressed in PT-expressing strains. We then performed competitive EMSAs and monitored PT frequencies to explain the contradictory observations, and the results showed that the PT modification protein complex DndCDE and TorR competitively bind to the *torCAD* regulatory region. As the *torCAD* operon encodes a TMAO reductase respiratory system, we monitored cell growth and found that competitive binding of DndCDE and TorR caused cell growth defects when respiration was based on TMAO. Considering these results, we proposed a competitive binding mechanism in which DndCDE competitively binds to the promoter region of *torCAD*, repressing *torCAD* transcription *in vivo.* However, one can envisage a scenario in which DndCDE binds and modifies the *tor* boxes, and TorR has a stronger affinity for the resulting PT-modified 5′-G_PS_TTC-3′/5′-G_PS_AAC-3′ sites, which might counteract the repressive effect of DndCDE proteins. However, this possibility was excluded because the transcription of *torCAD* in DndCDE-expressing Cerro 87 cells was comparable to that in DndC_C280S_DE-expressing HY1 cells.

Our observations in this study support the possibility that PT systems can modulate gene transcription in two ways: through the chemical structure of PT and through a competitive binding mechanism via Dnd proteins. In DNA methylation systems, utilizing the DNA methylation structure to regulate transcription is a common way. In eukaryotes, the methylation of CpG sites can impair the binding of transcription factors or recruit repressive methyl-binding proteins to inhibit gene transcription (2, 3). In prokaryotes, DNA methylation can interfere with transcription factors or RNA polymerase binding to DNA, thereby modulating gene expression (20). On the other hand, DNA modification enzymes that regulate transcription have also been observed in instances of eukaryotic DNA methylation. Several studies in mammals have shown that DNA methyltransferases (Dnmts) function as corepressors to silence gene expression upon interacting with histone deacetylases or other transcription factors (58–61). Nevertheless, in bacteria, no data have been reported to suggest that a DNA methylase itself directly participates in transcriptional regulation. Although several observations support competition between Dam methylase and transcription factors at overlapping 5′-GATC-3′ sites in DNA, such as the promoter regions of *agn43* and *papBA* (62, 63), this competition has not been reported to play a role in transcriptional regulation; however, the distinct methylation patterns resulting from competition have been suggested to modulate downstream gene transcription (10, 20, 21). Therefore, we are the first to report that a DNA modification enzyme in bacteria, the DNA PT modification protein complex DndCDE, participates in transcriptional regulation through a competitive binding mechanism in this study.

The *torCAD* operon encodes three proteins, TorC, TorA, and TorD, that enable the use of TMAO as a terminal electron acceptor for respiration (54, 64). According to a recent study, bacteria use TMAO for respiration both in the presence and absence of oxygen, but the expression profile of *torCAD* across the population is strikingly different under these two growth conditions (55). In aerobic cultures, the cellular levels of *torCAD* transcription are quite heterogeneous. In contrast, in anaerobic cultures, *torCAD* transcription is relatively homogeneous across populations (55, 65). This unique difference in expression patterns is presumed to serve as a bet-hedging strategy for bacteria, providing a fitness advantage when oxygen availability rapidly decreases (65). Based on our observation that both the PT modification and DndCDE proteins interfere with TorR binding to DNA, a conceivable speculation is that PT contributes to the adaptation of bacteria to rapid aerobic-to-anaerobic transitions. Research in this area is worth further exploration, as it may elucidate whether PT plays a role in bacterial environmental adaptation.

TorR is a transcriptional regulator in the TCS subfamily (66). The DNA binding function of the proteins in this family is generally driven by their CTD, which contains a helix-turn-helix (HTH) structure typically responsible for DNA binding (67). The PT-binding preference of TorR is reminiscent of ScoMcrA, a type IV restriction endonuclease that specifically recognizes PT through a sulfur-binding domain (SBD) (68, 69). The SBD contains a hydrophobic cavity to specifically recognize the PT sulfur (68). Although no significant sequence similarity was identified between TorR and SBD, we speculate that the increased binding of TorR to PT-modified DNA is attributed to altered hydrophobic and/or electrostatic interactions between certain residues in the CTD of TorR and PT sulfur.

In summary, our study shows that the PT-based Dnd system in S. enterica participates in the transcriptional regulation of the *torCAD* operon. *In vitro*, the PT structure increases the binding of TorR, a transcription activator of *torCAD*, to *tor* boxes. *In vivo*, the PT modification-related protein DndCDE complex represses the transcription of *torCAD* through a competitive binding mechanism. The lower transcription level of *torCAD* caused by competitive binding leads to growth defects in strains that use TMAO for respiration. These findings provide a deeper understanding of the characteristics of PT modification in the protein–DNA substrate recognition process and expand our knowledge of the molecular mechanisms underlying PT system-modulated gene regulation.

## MATERIALS AND METHODS

### Reagents, strains, plasmids, and cell culture.

Streptavidin magnetic beads were purchased from New England Biolabs (NEB). Proteomic analysis was performed at Beijing Genomics Institute (BGI, Shenzhen, China). TMAO was purchased from Sigma (Sigma–Aldrich Company, Ltd., Dorset, United Kingdom). d-Serine and sodium formate were purchased from Macklin (Shanghai, China). Oligonucleotides were synthesized at GenScript (Nanjing, China). The primers used in this study are listed in Table S1 in the supplemental material. All strains and plasmids used in this study are listed in Table S2. Strains were cultured in Luria broth (LB) or modified M9 minimum medium (glycerol was used as a carbon source) at 37°C or 28°C. Aerobic cultures were established in conical vials with aeration by shaking at 220 rpm in an incubator. For anaerobic culture, culture tubes were filled completely with culture medium, inoculated, and sealed tightly with threaded caps prior to standing incubation. When necessary, ampicillin, kanamycin, and chloramphenicol were added at final concentrations of 100 μg/mL, 50 μg/mL, and 25 μg/mL, respectively.

### Pulldown assay.

The Cerro 87 strain was inoculated in LB medium and cultured at 28°C overnight; the next day, it was diluted 1:50 with 300 mL of fresh LB medium and grown to an optical density at 600 nm (OD_600_) of ∼1.0. The cells then were harvested by centrifugation at 5,000 × *g* for 15 min. The cell pellet was washed with phosphate-buffered saline (PBS) three times and resuspended in 12 mL of suspension buffer (10 mM Tris-HCl, 30 mM NaCl, 60 mM KCl, 10 mM MgCl_2_, 1 mM EDTA, and 1 mM phenylmethylsulfonyl fluoride [PMSF], pH 7.2). The resuspended cells were lysed by sonication on ice and centrifuged at 17,000 × *g* for 30 min at 4°C. The supernatant was then transferred to a new tube, and the protein concentration was measured using the Bradford method (70). The supernatant sample was then diluted with suspension buffer to a final concentration of 4 mg/mL.

DNA substrates B12 and B34 were prepared by annealing oligonucleotides B1 and B2, B3 and B4, respectively. Fifty microliters of streptavidin magnetic beads (which bind 100 pmol of DNA) was used in each sample. First, the beads were washed with oligonucleotide binding buffer (0.5 M NaCl, 20 mM Tris-HCl, and 1 mM EDTA, pH 7.5) three times and resuspended in 500 μL of oligonucleotide binding buffer, and then 250 pmol DNA substrate (5 μL) was added and incubated with the beads on a rotator for 1 h at room temperature to ensure that the beads were completely bound to the DNA substrate. The beads then were washed three times with oligonucleotide binding buffer on a magnetic separator to remove unbound DNA substrate, and then 500 μL of cell lysate was added to the beads and incubated on a rotator at 4°C for 4 h to allow the proteins in the cell lysate to bind to the DNA substrate on the beads. The beads were washed with suspension buffer 5 times on a magnetic separator to remove nonspecifically bound proteins. The bound proteins were subsequently released by adding SDS-PAGE protein loading buffer, incubated at 95°C for 10 min, and then centrifuged at 15,000 × *g* for 10 min. The supernatant was used for protein identification and quantification. The full data set showing all the pulled-down proteins detected by the proteomic analysis is presented in Data Set S1.

### Proteomic analysis.

The pulled-down proteins were separated on 10% SDS-PAGE gels, and electrophoresis was stopped when the bromophenol blue band reached ∼1 cm below the stacking gel. The in-gel trypsin digestion of proteins and peptide extraction was performed as described by Hu et al. (71).

The digested peptides were desalted using a Strata X column (Phenomenex), vacuum dried, and then resuspended in 200 μL of buffer A (2% acetonitrile [ACN] and 0.1% formic acid [FA]). After centrifugation at 20,000 × *g* for 10 min, 10 μL of supernatant (containing approximately 5 μg of protein) was loaded with an autosampler onto a 2-cm C_18_ trap column of an LC-20AD nano-high-performance liquid chromatography system (Shimadzu, Kyoto, Japan). The peptides were then eluted onto a 10-cm analytical C_18_ column (inner diameter of 75 μm) packed in-house. The samples were loaded at a rate of 8 μL/min for 4 min, and then a 44-min gradient was run with 2% to 35% B (98% ACN, 0.1% FA) at a rate of 300 nL/min, followed by 2 min with a linear gradient to 80% B, maintained with 80% B for 4 min, and finally returned to 2% B in 1 min. The peptides were subjected to nanoelectrospray ionization followed by MS/MS with a Q Exactive spectrometer (Thermo Fisher Scientific, San Jose, CA). Intact peptides were detected in the Orbitrap at a resolution of 70,000. Peptides were selected for MS/MS using the high-energy collision dissociation operating mode with a normalized collision energy setting of 27.0; ion fragments were detected in the Orbitrap at a resolution of 17,500. A data-dependent procedure that alternated between one MS scan followed by 15 MS/MS scans was applied for the 15 most abundant precursor ions exceeding a threshold ion count of 20,000 in the MS survey scan, with a subsequent dynamic exclusion duration of 15 s. The electrospray voltage applied was 1.6 kV. Automatic gain control (AGC) was used to optimize the spectra generated by the Orbitrap. The AGC target for the full MS scan was 3e^6^ and 1e^5^ for MS2. For MS scans, the *m/z* scan range was 350 to 2,000. For MS2 scans, the *m/z* scan range was 100 to 1,800.

Raw data files acquired from the Orbitrap were converted into MGF files using Proteome Discoverer 1.2 software (PD 1.2; Thermo), and the MGF file was searched. Proteins were identified using the Mascot search engine (version 2.3.02; Matrix Science, London, United Kingdom) by searching against a database containing 4,511 sequences. For protein identification, a mass tolerance of 20 ppm was permitted for intact peptide masses and 0.1 Da for fragmented ions. Gln→pyro-Glu (N-terminal Q), oxidation (M), and deamidation (NQ) were set as potential variable modifications, and carbamidomethyl (C) was set as a fixed modification. Peptide charge states were set to +2 and +3. Only peptides with significance scores (≥20) at a 99% confidence interval obtained from a Mascot probability analysis greater than “identity” were counted as identified to reduce the probability of false peptide identification. Each confident protein identification involved at least one unique peptide.

### EMSA.

The purified TorR protein or DndCDE complex was incubated with 50 nM DNA substrates, i.e., box1 or box2, in 20 μL of binding buffer (20 mM Tris-HCl, pH 8.0, 50 mM KCl, 10 mM MgCl_2_, 5 mM EDTA, 10 mM dithiothreitol [DTT], and 5% glycerol). The mixtures were incubated at 28°C for 30 min before they were loaded onto native polyacrylamide gels (9% gel for TorR protein and 4% gel for DndCDE complex). Electrophoresis was performed at 4°C with 0.5× Tris-borate-EDTA buffer. The gel was then stained with GelRed and photographed with a Gel Doc XR+ (Bio-Rad) imaging system. A kinetic curve was drawn according to the decrease in grayscale of the nonmigrated DNA band.

### Protein expression and purification.

The *torR* gene was PCR amplified using Cerro 87 genomic DNA as a template and cloned into pGEX-6p-1 that had been digested with BamHI and SalI, generating pWHU3453. For TorR_NTD_ and TorR_CTD_, the N terminus (M1-R131) and C terminus (P139-Y242) of the *torR* gene were PCR amplified and cloned into the NdeI-EcoRI and NcoI-XhoI sites of pET28a, generating pWHU3458 and pWHU3459, respectively. For DndCDE complex expression, the *dndCDE* operon genes were amplified together using Cerro 87 genomic DNA as a template with a 6× His tag fused to the N terminus of DndC and cloned into the NdeI-BamHI site of pET28a, generating pWHU3454. These plasmids were then transformed into competent E. coli BL21(DE3) cells. The transformants were first grown in LB medium at 37°C with the respective antibiotics to an OD_600_ of 1.0, and then the temperature was changed to 16°C and the cells were induced with 0.2 mM isopropyl-β-d-thiogalactopyranoside (IPTG) for another 20 h. The cells were harvested by centrifugation at 5,000 × *g* for 10 min. For TorR purification, the cells were resuspended in lysis buffer (20 mM Tris-HCl, pH 8.0, and 150 mM NaCl), lysed by sonication on ice, and centrifuged at 15,000 × *g* for 30 min. The supernatant was recovered, filtered through a 0.45-μm microfiltration membrane (Millipore), and subsequently loaded onto a glutathione-*S*-transferase (GST)-agarose (Yeason, Shanghai, China) self-packed gravity column (2 mL) preequilibrated with lysis buffer. The protein was eluted with 10 mL of elution buffer (lysis buffer supplemented with 20 mM glutathione [GSH]). One milliliter of eluate was collected as one fraction and analyzed on 10% SDS-PAGE gels; the fractions containing the target protein were recovered and pooled. The protein was concentrated, and the buffer was replaced with stock buffer (20 mM Tris-HCl, pH 8.0, 150 mM NaCl, and 10% glycerol) with an Amicon Ultra centrifugal filter (10,000 molecular weight cutoff; Millipore). For TorR_NTD_ and TorR_CTD_ purification, cells were harvested and lysed using the same procedure for purifying TorR, but nickel nitrilotriacetic acid (Ni-NTA) affinity chromatography with a self-packed gravity column (2 mL) and lysis buffer supplemented with a gradient of 40, 80, 110, 300, and 500 mM imidazole (5 mL each gradient) were used for TorR_NTD_ and TorR_CTD_. Fractions containing the target protein were recovered, concentrated to a 1-mL volume and subjected to size exclusion chromatography with an ÄKTA protein liquid chromatography system (GE Life Sciences) using a GE HiLoad Superdex 16/600 200 pg column (GE Life Sciences) preequilibrated with lysis buffer. Fractions containing the target protein were recovered. The protein was concentrated, and the buffer was replaced with stock buffer by centrifugation using an Amicon Ultra centrifugal filter (10,000 MWCO; Millipore) at 4°C and 4,500 × *g*. Because DndCDE form a complex, these proteins were copurified with His-tagged DndC as described by Pu et al. (72).

### β-Galactosidase assay.

Through fusion PCR, 122-bp, 340-bp, and 225-bp segments of the *torCAD*, *fdhF*, and *dsdXA* promoters were fused to the promoterless *lacZ* gene. The P*_torCAD_*-*lacZ*, P*_fdhF_*-*lacZ*, and P*_dsdXA_*-*lacZ* fusions were then cloned into the BamHI-HindIII, SalI-HindIII, and SalI-HindIII sites of pACYC184, generating pWHU3443, pWHU3441, and pWHU3442, respectively. These plasmids were then transfected into Cerro 87 and XTG103 cells. The transformants were grown in LB medium supplemented with chloramphenicol at 28°C overnight and then diluted 1:100 with fresh LB medium supplemented with TMAO (20 mM), sodium formate (30 mM), or d-serine (500 μg/mL). When the cells reached the mid-log phase of growth, β-galactosidase activity was measured using the method described by Zhang et al. (73, 74). For P*_fdhF_*-*lacZ*, β-galactosidase activity was measured under anaerobic conditions, as the transcription of *fdhF* is induced under anaerobic growth conditions. For P*_torCAD_*-*lacZ*, β-galactosidase activity was measured in strains cultured under both aerobic and anaerobic conditions.

### qRT-PCR.

The relative abundance of *torC* mRNA was measured using quantitative real-time PCR (qRT-PCR). Bacteria were cultured at 28°C in LB medium supplemented with 20 mM TMAO under anaerobic conditions until they reached the mid-log phase (OD_600_ 0.8 to ∼1.0). For strains harboring pWHU3450 and pWHU3455, 0.4% glucose or 0.4% arabinose was added to repress and induce *dnd* operon expression, respectively. The cells were then harvested, and total RNA was extracted using a bacterial RNA kit (Omega). One microgram of RNA was then treated with DNase I (RNase free) and reverse transcribed using a RevertAid first-strand cDNA synthesis kit (Thermo Scientific). Specific primers amplifying products of 147 bp (*torC*) or 120 bp (the housekeeping gene *gapA*, which was used for normalization) were designed using Primer 6 software. Primers 5′-ACCTGTCGTTCCTGCCATAA-3′ and 5′-GCTCATATCCGGTAGCTGGT-3′ were used to amplify the *torC* gene; primers 5′-GAAGGCCAGGACATCGTTTC-3′ and 5′-AGTCGCGTGAACAGTGGTCA-3′ were used to amplify the *gapA* gene. Real-time PCR was performed using SYBR green master mix (Vazyme) and a CFX Connect real-time system (Bio-Rad) to determine the threshold cycle (*C_T_*). The comparative threshold cycle (2^−ΔΔ^*^CT^*) method was used to compare relative mRNA levels.

### Quantification of the PT modification.

E. coli DH10B harboring pWHU3451 and pWHU3452 was grown anaerobically at 37°C in LB medium (supplemented with 20 mM TMAO) containing 0.4% glucose to repress *torR* expression or 0.4% arabinose to induce *torR* expression. When cells reached the mid-log growth phase (OD_600_ of ∼0.8), they were harvested and the plasmid was extracted, hydrolyzed, and dephosphorylated, as described by Lai et al. (75). LC-MS/MS was performed to quantify PT-modified dinucleotides as described previously (23, 75). For PT quantification, 5 pmol d(G_PS_A) S_P_ was added to each sample as a reference, and the relative PT modification abundances were compared.

### Monitoring cell growth.

Growth was evaluated anaerobically at 28°C to monitor the growth of strains solely dependent on TMAO respiration. Notably, under anaerobic conditions, bacteria can grow by fermentation as well as anaerobic respiration; thus, a modified M9 medium containing a single, nonfermentable carbon source (glycerol) was used for growth. Bacteria were cultured in LB medium overnight, washed once with modified M9 medium (nonfermentable glycerol [0.4%] used as a carbon source), and then diluted 1:100 with fresh modified M9 medium supplemented with 20 mM TMAO. When needed, 0.1 mM arabinose was added to induce expression. At the indicated times, 200 μL of cell culture was sampled and placed in a well of a transparent 96-well plate, and the OD_600_ was measured using a microplate reader (Multiskan GO; Thermo Scientific). Aerobically cultured cells were used as controls because these cells grew by aerobic respiration and no longer relied on TMAO respiration.

### LC-MS/MS quantification of TMAO and TMA levels.

In the TMAO respiratory pathway, TMAO is reduced to TMA by TMAO reductase. LC-MS/MS was used to detect and quantify TMAO and TMA levels. Bacteria were cultured using the procedure for monitoring cell growth described above. When indicated, 0.1 mM arabinose was added to induce expression. At the indicated times, 200 μL of cell culture was sampled and centrifuged at 15,000 × *g* for 10 min; the supernatant was then ultrafiltrated by centrifugation with a Pall Nanosep centrifugal device with Omega membrane–10K (Life Science) and then diluted 10 times with double-distilled water. A Thermo Hypersil GOLD aQ column coupled to a Thermo TSQ quantum access MAX mass spectrometer was used for detection. The mobile phase consisted of a mixture of 10 mM ammonium acetate (pH 3.0) as solvent A and ACN as solvent B. A mobile-phase proportion consisting of 60:40 (A:B) was used for detection. The injection volume was 10 μL. The flow rate was 0.1 mL/min. Electrospray ionization (ESI) was used in positive mode. *m/z* 76.0→58.2, 76.0→42.3, and 76→30.1 were used to monitor precursor-product ion transitions of TMAO, and 60.0→44.4 was used to monitor TMA. The metabolic rate of TMAO was calculated as

$$\frac{\text{peak area of TMA }(60.0\to 44.4)}{\text{peak area of TMAO }(76.0\to 58.2)\;+\text{ peak area of TMA }(60.0\to 44.4)}$$

### Data availability.

The mass spectrometry proteomics data have been deposited in the ProteomeXchange Consortium via the PRIDE (76) partner repository with the data set identifier PXD031869.
