# Supplementary material for: Involvement of the DNA Phosphorothioation System in TorR Binding and Anaerobic TMAO Respiration in Salmonella enterica
Source: mBio. 2022 Apr 14;13(3):e00699-22. doi: 10.1128/mbio.00699-22 (PMC9239176; doi:10.1128/mbio.00699-22)
Supplement: TABLE S1 [file mbio.00699-22-s0002.docx]

**TABLE S1** Primer sequences used in this study

| **Primers** | **Sequences (5’-3’)** |
| --- | --- |
| **Construction of the B12 probe** | |
| B1 | biotin-GGCCCACTACGTG_PS_AACCATCACCCTAATCA |
| B2 | TGATTAGGGTGATGG_PS_TTCACGTAGTGGGCC |
| **Construction of the B34 probe** | |
| B3 | biotin-GGCCCACTACGTGAACCATCACCCTAATCA |
| B4 | TGATTAGGGTGATGGTTCACGTAGTGGGCC |
| **Construction of pWHU3441** | |
| *fdhF*pro-*lacz*-1 | AAACGCGTCGACTGCCGCGGCCCGTTCTATCC |
| *fdhF*pro-*lacz*-2 | GAATCCGTAATCATGGTCATCGGTTTCGCTCCAGTTAATC |
| *fdhF*pro-*lacz*-3 | GATTAACTGGAGCGAAACCGATGACCATGATTACGGATTC |
| *fdhF*pro-*lacz*-4 | CCCAAGCTTTTATTTTTGACACCAGACCA |
| **Construction of pWHU3442** | |
| *dsdXA*pro-*lacz*-1 | AAACGCGTCGACGGCAACCTCAGTCATCTTTT |
| *dsdXA*pro-*lacz*-2 | GAATCCGTAATCATGGTCATATCTCACCTACCCTGTGATT |
| *dsdXA*pro-*lacz*-3 | AATCACAGGGTAGGTGAGATATGACCATGATTACGGATTC |
| *dsdXA*pro-*lacz*-4 | CCCAAGCTTTTATTTTTGACACCAGACCA |
| **Construction of pWHU3443** | |
| *torCAD*pro-*lacz*-1 | CGGGATCCGCGGTCTCTTTCTGTTCATATC |
| *torCAD*pro-*lacz*-2 | ATCCGTAATCATGGTCATAATAGCCCCTGTAATTAT |
| *torCAD*pro-*lacz*-3 | ATAATTACAGGGGCTATTATGACCATGATTACGGAT |
| *torCAD*pro-*lacz*-4 | CCCAAGCTTTTATTTTTGACACCAGACCA |
| **Construction of pWHU3450 and pWHU3455** | |
| *dndCDE*-pBAD-F | AGCAGGAGGAATTCACCATGGGCATGAGTAAATTAGTTCAGGCC |
| *dndCDE*-pBAD-R | GCCTGCAGGTCGACTCTAGACTATATATTCTTTGAAAAAT |
| **Construction of pWHU3451 and pWHU3452** | |
| pBAD*torR*-1238-F | ACTCACCGCGGATCGATGCATAATGTGCCTG |
| pBAD*torR*-1238-R | ATGCGAGCTCCTACTTGTCATCGTCATCCTTGT |
| **Construction of pWHU3453** | |
| *torR*-pGEX-F | CGCGGATCCATGAACAGATATGAACAGAA |
| *torR*-pGEX-R | ACGCGTCGACTCAATACACCTCCGAGGCT |
| **Construction of pWHU3454** | |
| *dndCDE*-pET-F | GGAATTCCATATGATGAGTAAATTAGTTCAGG |
| *dndCDE*-pET-R | CGGGATCCCTATATATTCTTTGAAAAAT |
| **Construction of pWHU3458** | |
| *torR*-NTD-F | GGAATTCCATATGAACAGATATGAACAGAA |
| *torR*-NTD-R | CCGGAATTCTTAACGCCACAAAAGATTTTTCA |
| **Construction of pWHU3459** | |
| *torR*-CTD-F | CATGCCATGGGCCCGCAAAATGCCAGTGAAAAC |
| *torR*-CTD-R | CCGGAACTCGAGATACACCTCCGAGGCTAAAAA |
