# Supplementary figures and images for: Involvement of the DNA Phosphorothioation System in TorR Binding and Anaerobic TMAO Respiration in Salmonella enterica
Source: mBio. 2022 Apr 14;13(3):e00699-22. doi: 10.1128/mbio.00699-22 (PMC9239176; doi:10.1128/mbio.00699-22)

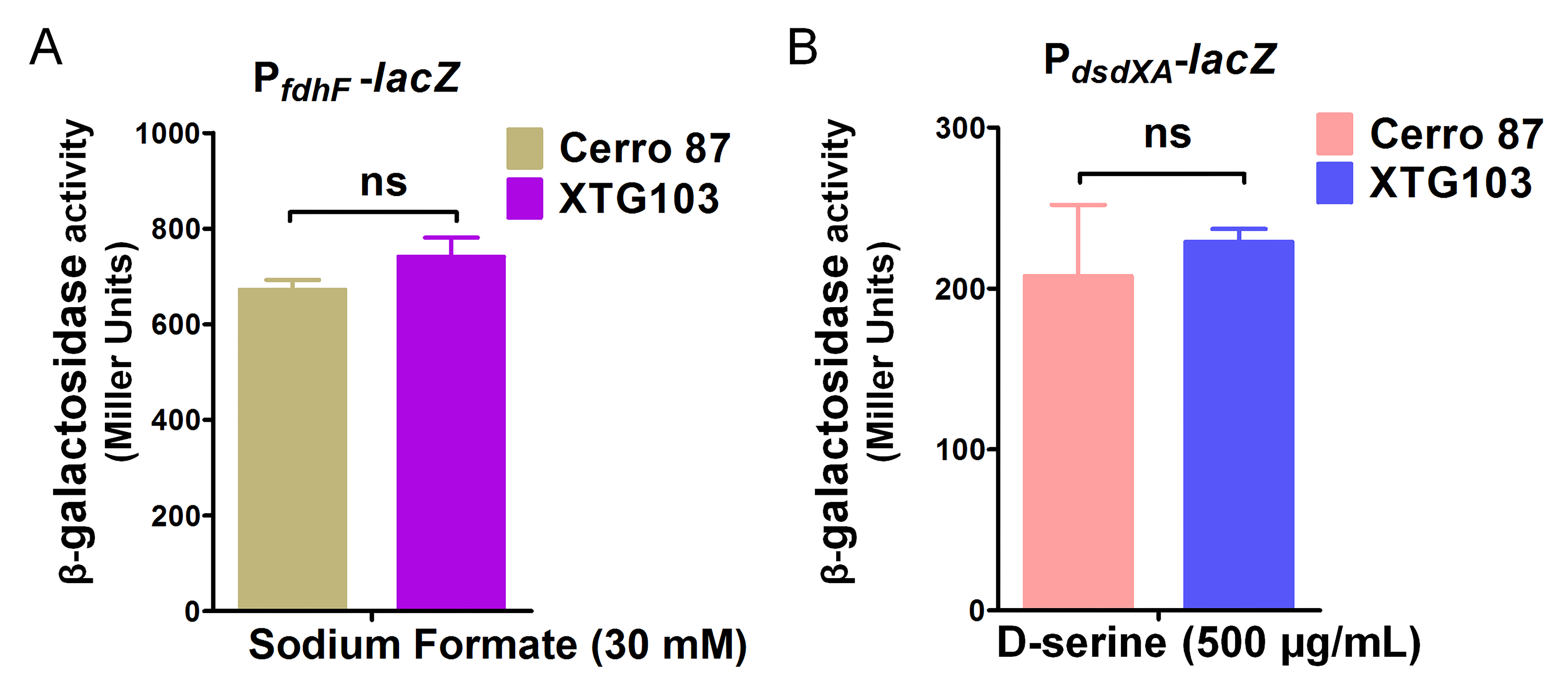

Supplement: FIG S1 [file mbio.00699-22-s0001.tif]
