# Supplementary material for: Involvement of the DNA Phosphorothioation System in TorR Binding and Anaerobic TMAO Respiration in Salmonella enterica
Source: mBio. 2022 Apr 14;13(3):e00699-22. doi: 10.1128/mbio.00699-22 (PMC9239176; doi:10.1128/mbio.00699-22)
Supplement: TABLE S2 [file mbio.00699-22-s0003.docx]

**TABLE S2** Strains and plasmids used in this study

| **Strains and plasmids** | **Characteristics** | **Source or reference** |
| --- | --- | --- |
| ***S. enterica strains*** |  |  |
| Cerro 87 | Wild type | (1) |
| XTG103 | Cerro 87 derivative, *dndBCDE-dndFGH* in-frame deletion mutant | (1) |
| CX1 | Cerro 87 derivative, *dndFGH* in-frame deletion mutant | This work |
| HY1 | CX1 derivative, expressing DndC_C280S_DE | This work |
| ***E. coli*** |  |  |
| DH10B | F-*mcr*AΔ(*mrr*-*hsd*RMS-*mcr*BC) ϕ80*lac*ZΔM15Δ*lac*X74 *rec*A1 *end*A1 *ara*D139 Δ(*ara*, *leu*)7697 *gal*E15 *gal*K λ-*rps*L *nup*G | TransGene Biotech |
| **Plasmids** |  |  |
| pACYC184 | Cloning vector, Cm^R^, Tc^R^ | (2) |
| pBluescript II SK(+) | Cloning vector, Amp^R^ | (3) |
| pBAD24 | Cloning vector containing an arabinose-inducible pBAD promoter, Amp^R^ | (4) |
| pET28a | Cloning vector, Kan^R^ | Novagen |
| pGEX-6P-1 | Cloning vector, Amp^R^ | Amersham |
| pZWHJ002 | pXMJ19 derivative containing promoterless *lacZ*, Cam^R^ | (5) |
| pJTU1238 | *dndBCDE* from *S. enterica* cloned in pBluescript II SK(+) | (1) |
| pWHU3441 | pACYC184 derivative with a 3415 bp P*_fdhF_*-*lacZ* fragment cloned into the BamHI-HindIII site | This work |
| pWHU3442 | pACYC184 derivative with a 3300 bp P*_dsdXA_*-*lacZ* fragment cloned into the SalI-HindIII site | This work |
| pWHU3443 | pACYC184 derivative with a 3197 bp P*_torCAD_*-*lacZ* fragment cloned into the SalI-HindIII site | This work |
| pWHU3450 | pBAD24 derivative with the *dndCDE* operon cloned under the control of pBAD promoter | This work |
| pWHU3451 | pJTU1238 derivative with the *torR* gene cloned under the control of pBAD promoter and 5 direct repeats of the regulatory region of the *torCAD* operon | This work |
| pWHU3452 | pJTU1238 derivative with the *torR* gene cloned under the control of pBAD promoter | This work |
| pWHU3453 | pGEX-6P-1 derivative expressing TorR of Cerro 87, expression vector | This work |
| pWHU3454 | pET28a derivative expressing DndCDE of Cerro 87, expression vector | This work |
| pWHU3455 | pBAD24 derivative with the *dndC_C280S_DE* operon cloned under the control of pBAD promoter | This work |
| pWHU3458 | pET28a derivative expressing TorR_NTD_(M1-R131), expression vector | This work |
| pWHU3459 | pET28a derivative expressing TorR_CTD_(P139-Y242), expression vector | This work |

**Reference**

1. Xu T, Yao F, Zhou X, Deng Z, You D. 2010. A novel host-specific restriction system associated with DNA backbone S-modification in Salmonella. Nucleic acids research 38:7133-7141.

2. Rose RE. 1988. The nucleotide sequence of pACYC184. Nucleic acids research 16:355.

3. Alting-Mees M, Short J. 1989. pBluescript II: gene mapping vectors. Nucleic acids research 17:9494.

4. Guzman L-M, Belin D, Carson MJ, Beckwith J. 1995. Tight regulation, modulation, and high-level expression by vectors containing the arabinose PBAD promoter. Journal of bacteriology 177:4121-4130.

5. Chao H, Zhou N-Y. 2014. Involvement of the global regulator GlxR in 3-hydroxybenzoate and gentisate utilization by Corynebacterium glutamicum. Appl Environ Microbiol 80:4215-4225.
